# Notes and News

**Published:** 1895-12-07

**Authors:** 


### Notes and News.

(Collected and Communicated.)

According to Dr. Dubois, who lectured recently at the Anthropological Institute, the missing link has been found. He illustrated his contention by showing fossil remains found in Java, which he held showed skeleton remains of a type between that of apes and men.

The secretary of the North-Eastern Hospital for Children writes: In reference to the appeal for this hospital appearing in a recent issue of your valuable paper, a governor of the institution has promised to give £25 provided nine other similar donations are obtained by the 31st inst.

The Duchess of Teck has given her countenance to a scheme for establishing a private hospital and home of rest for members of the theatrical profession. The scheme inaugurated by Mrs. Willard some time ago has not been put into effect owing to lack of funds, but an appeal to the whole profession is now to be made.

The medical officers of the Camberwell Provident Dispensary have written to the *British Medical Journal* protesting against the proposed scheme to erect a general hospital in the locality. They consider such action entirely unnecessary, and that their dispensary is able to minister to the wants of the sick poor of Camberwell, with the aid of Guy's and St. Thomas's Hospital.

A meeting was held recently at Oxford on behalf of the Oxford bed at the hospital at Jerusalem, at which the necessity of a new hospital was urgently put forward. The building has been already commenced, and £3,000 is required to complete it free of debt. The present building is in a bad and insanitary position. Oxford maintains one bed, and Cambridge three at the hospital, at a cost of £20 per annum per bed.

A cottage hospital has been opened at Pershore, in Worcestershire, by the Countess of Coventry. The hospital owes its establishment to the generosity of the late Mr. Ganderton, who bequeathed £580 to form the nucleus of a fund for the purpose. The whole cost of £1,200 has been contributed. The building is of red brick. On the ground floor is a ward for male patients, whilst a temporary one has been arranged for female patients on the first floor. Two beds for men and two for women are now provided.

As a warning to other institutions the secretary of the Cancer Hospital, Fulham Road, asks us to state that a robbery from the nurses' dormitories has been effected at his hospital by a man who called there after the workpeople employed in installing the electric light had left for the day, and stated he was engaged on the arrangement of switches and so obtained access to all parts of the building, and being finally left to himself succeeded in departing with various articles of value and some money. The man appeared thoroughly acquainted with the work, and thus deceived the officials.

The case tried before Mr. Justice Kekewich between the residents near Guildford and the Guildford, Goldalming, and Woking Joint Hospital Board, in which the former tried to restrain the Board from using a site selected for a small-pox hospital, brought many interesting points into discussion, and the action will be useful as a precedent on future occasions. Dr. Thorne Thorne gave evidence before the Court that in his opinion contagion from small-pox might be conveyed in the atmosphere under certain conditions as far as a mile, but that vaccinated children and adults revaccinated were practically immune from the disease. Mr. Justice Kekewich, keeping strictly to the main contention of the plaintiffs, that the hospital would be a danger to the locality, gave judgment for the defendants, as he held that the theory of contagion was not sufficiently established to warrant the assumption that a small-pox establishment on a site of two acres, if properly administered, would so act. At the same time, he held the Board pledged not to utilise the present cottage, described as ruinous, or any other building, save a properly constructed one, as a small-pox hospital.

The temerity of some people in regard to the charities in which they are interested is astonishing at times. Thus, Mr. Cane Fowler, in seconding the adoption of the report at the annual meeting of the Royal Hospital for Incurables, is stated in the *Daily Chronicle* of 30th ultimo to have declared, "Piles of papers were supplied to the House of Lords making charges against it, but when they asked for evidence it was refused them. Although these accusations were spread broadcast, no one had dared to come forward with an attempt to establish them." We should like to ask Mr. Cane Fowler and the committee of the Royal Hospital for Incurables to print the evidence taken at the offices of the Royal Hospital for Incurables by the committee of which Lord Aberdeen was president after the Lords' Committee's report was published. They must be perfectly well aware that the management of this institution can never be regarded as satisfactory so long as it is conducted without the presence of a resident medical superintendent and a staff of efficiently trained male and emale nurses acting under his direction.

